# 1-(3-Oxo-3-phenyl­prop­yl)piperidinium chloride

**DOI:** 10.1107/S1600536813029887

**Published:** 2013-11-06

**Authors:** Venkatramu Anuradha, S. Madan Kumar, B. P. Siddaraju, N. K. Lokanath, P. Nagendra

**Affiliations:** a Department of Physics, Dr M. G. R. Educational and Research Institute, Maduravoyal, Chennai, India; b Department of Studies in Physics, University of Mysore, Manasagangotri, Mysore 570 006, India; c Department of Chemistry, BET Academy of Higher Education, Bharathi College, Bharthi Nagara, Mandya 571 422, India

## Abstract

In the title salt, C_14_H_20_NO^+^·Cl^−^, the piperidine ring adopts a chair conformation. In the crystal, the cations and anions are linked by classical N—H⋯Cl hydrogen bond and weak C—H⋯Cl and C—H⋯O hydrogen bonds; the C—H⋯O hydrogen bonds exhibit *R*_2_^2^(14) ring motifs while the C—H⋯Cl hydrogen bonds link the mol­ecules into chains along the *a*-axis direction. π–π stacking is observed between parallel phenyl rings of adjacent cations, the centroid–centroid distance being 3.8164 (15) Å.

## Related literature

For the synthesis and biological activity of piperidine derivatives, see: Vartanyan (1984 ▶). For standard bond lengths, see: Allen *et al.* (1987 ▶). For hydrogen-bond motifs see: Bernstein *et al.* (1995 ▶). For puckering parameters, see: Cremer & Pople (1975 ▶).
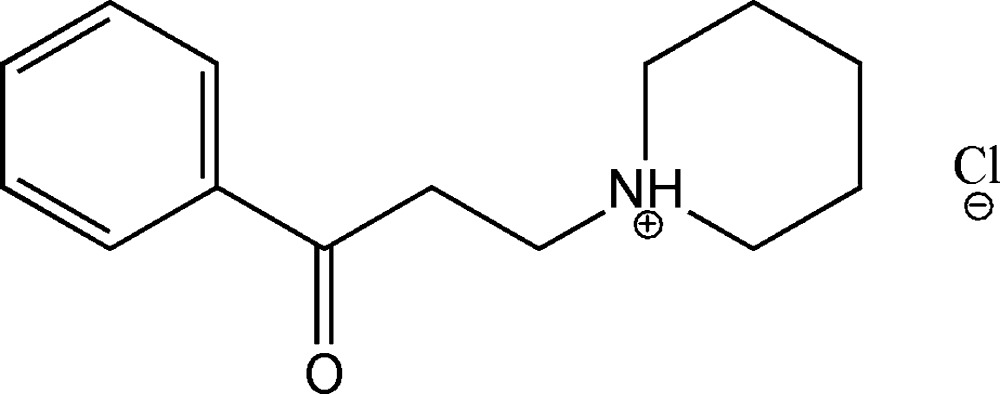


## Experimental

### 

#### Crystal data

C_14_H_20_NO^+^·Cl^−^
*M* *_r_* = 253.76
Monoclinic, 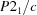

*a* = 11.2936 (13) Å
*b* = 12.0531 (15) Å
*c* = 10.9650 (13) Å
β = 112.971 (5)°
*V* = 1374.2 (3) Å^3^
*Z* = 4
Cu *K*α radiation
μ = 2.33 mm^−1^
*T* = 296 K
0.23 × 0.22 × 0.21 mm

#### Data collection

Bruker X8 Proteum diffractometer
Absorption correction: multi-scan (*SADABS*; Bruker, 2013 ▶) *T*_min_ = 0.558, *T*_max_ = 0.614
7001 measured reflections
2217 independent reflections
1833 reflections with *I* > 2σ(*I*)
*R*_int_ = 0.057

#### Refinement

*R*[*F* ^2^ > 2σ(*F* ^2^)] = 0.052
*wR*(*F* ^2^) = 0.130
*S* = 1.09
2217 reflections
159 parameters
H atoms treated by a mixture of independent and constrained refinement
Δρ_max_ = 0.31 e Å^−3^
Δρ_min_ = −0.50 e Å^−3^

### 

Data collection: *APEX2* (Bruker, 2013 ▶); cell refinement: *SAINT* (Bruker, 2013 ▶); data reduction: *SAINT*; program(s) used to solve structure: *SHELXS97* (Sheldrick, 2008 ▶); program(s) used to refine structure: *SHELXL97* (Sheldrick, 2008 ▶); molecular graphics: *Mercury* (Macrae *et al.*, 2008 ▶); software used to prepare material for publication: *SHELXL97* and *Mercury*.

## Supplementary Material

Crystal structure: contains datablock(s) global, I. DOI: 10.1107/S1600536813029887/xu5748sup1.cif

Structure factors: contains datablock(s) I. DOI: 10.1107/S1600536813029887/xu5748Isup2.hkl

Supplementary material file. DOI: 10.1107/S1600536813029887/xu5748Isup3.cml

Additional supplementary materials:  crystallographic information; 3D view; checkCIF report

## Figures and Tables

**Table 1:** Hydrogen-bond geometry (Å, °)

*D*—H⋯*A*	*D*—H	H⋯*A*	*D*⋯*A*	*D*—H⋯*A*
N1—H11⋯Cl1^i^	0.95 (2)	2.15 (2)	3.0837 (18)	171 (2)
C4—H4⋯Cl1^ii^	0.93	2.82	3.745 (3)	172
C14—H14*B*⋯O1^iii^	0.97	2.45	3.249 (3)	139

Symmetry codes: (i) 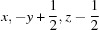; (ii) 
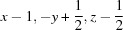
; (iii) 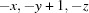.

**Fig. 1.**
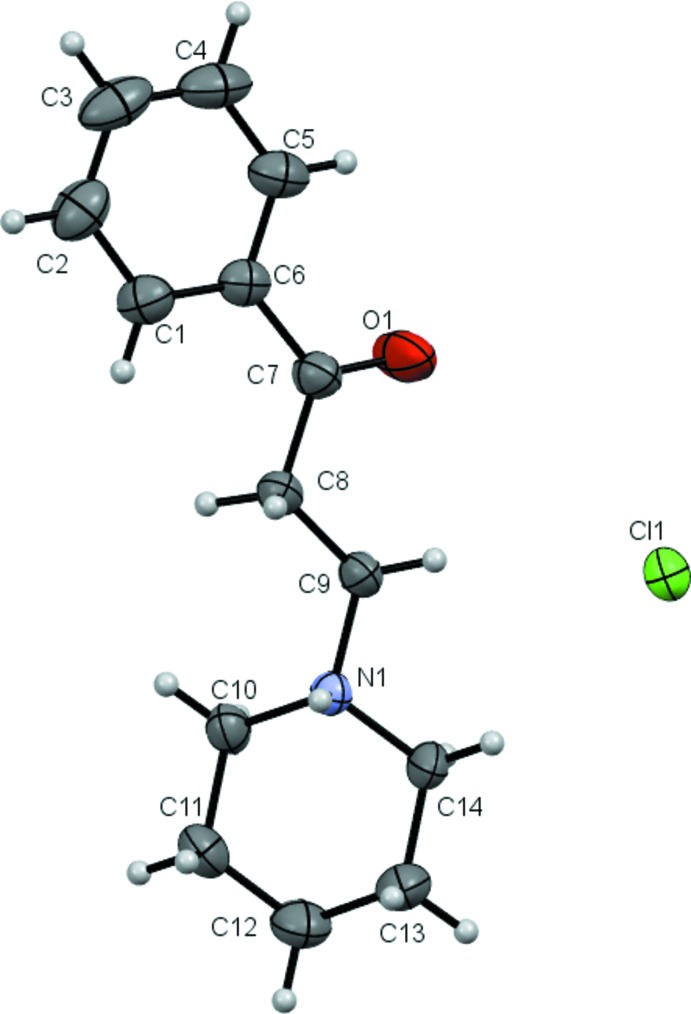
ORTEP diagram of the title compound with 50% probability ellipsoids.

**Fig. 2.**
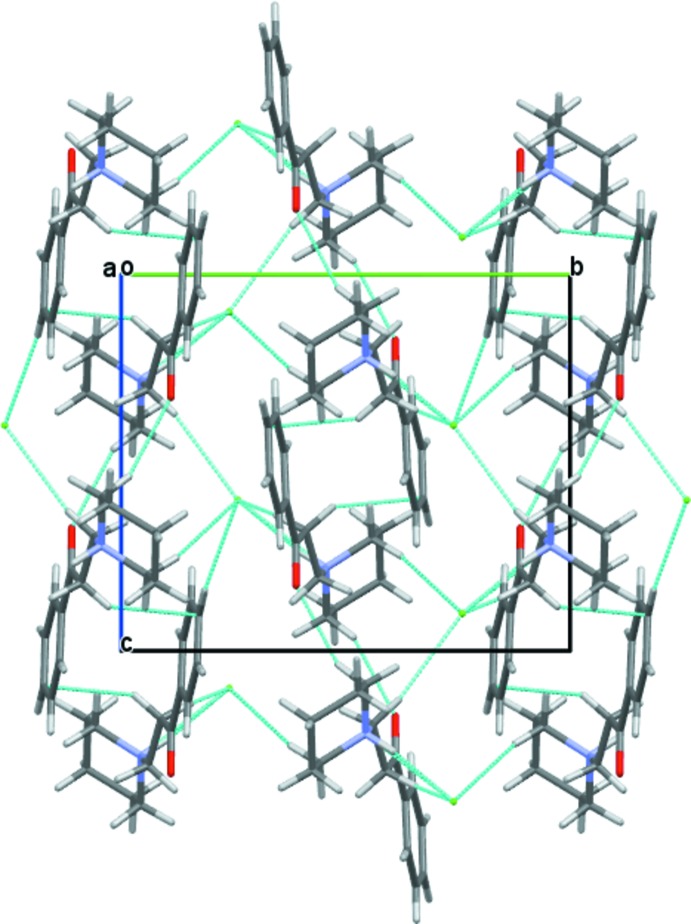
Packing diagram of molecule, viewed along the crystallographic a axis. Dotted lines indicate hydrogen bonds and short contacts involved.

## supplementary crystallographic information

### 1. Comment

The piperidine hydrochloride is used as an intermediate for the synthesis of pharmaceuticals such as haloperidol (neuroleptic drug used to treat patients with psychotic illnesses, extreme agitation, or Tourette's syndrome) and loperamide which is a synthetic piperidine derivative, is an effective drug against diarrhea resulting from gastroenteritis or inflammatory bowel disease (Vartanyan *et al.*, 1984).

The piperidine ring (N1/C10—C14) of the title compound (Fig. 1) adopts chair conformation. The puckering parameters of the piperdine ring are *Q* = 0.571 (2) Å, θ = 180.0 (2)° and φ = 19 (10)° (Cremer & Pople, 1975). The bond lengths and angles are in normal ranges (Allen *et al.*, 1987).

The molecules are packed along *a* axis with inter molecular hydrogen bonds are shown in Figure 2. Bond lengths and angles of intermolecular hydrogen bonds (N1—H11···Cl1, C14—H14B···O1 and C4—H4···Cl1) are listed in table 1. Also, N···Cl intercontacts with a distance of 3.084 Å is observed. The C14—H14B···O1, exhibits *R*^2^_2_(14) ring motifs (Bernstein *et al.*, 1995). The molecules are connected by infinite one dimensional chains along *a* axis by C4—H4···Cl1 hydrogen bonds. In addition, π···π interactions exists between phenyl rings *Cg*2···*Cg*2 with a distance of 3.8164 (15) Å, where *Cg*2 is C1/C2/C3/C4/C5/C6. Overall crystal structure of the title molecule exhibits three dimensional architecture.

### 2. Experimental

Single crystals (block) were obtained from slow evaporation of a solution of ethylacetate (m.p.:410–413 K).

### 3. Refinement

The H11 atom is located in a difference Fourier map and refined isotropically. Other H atoms were fixed geometrically (C—H= 0.93–0.96 Å) and allowed to ride on their parent atoms with *U*_iso_(H) = 1.5*U*_eq_ for methyl H atom and 1.2*U*_eq_(C) for other H atoms.

### Crystal data

**Table:**

C_14_H_20_NO^+^·Cl^−^	*F*(000) = 544
*M**_r_* = 253.76	*D*_x_ = 1.227 Mg m^−^^3^
Monoclinic, *P*2_1_/*c*	Cu *K*α radiation, λ = 1.54178 Å
Hall symbol: -P 2ybc	Cell parameters from 2217 reflections
*a* = 11.2936 (13) Å	θ = 4.3–64.4°
*b* = 12.0531 (15) Å	µ = 2.33 mm^−^^1^
*c* = 10.9650 (13) Å	*T* = 296 K
β = 112.971 (5)°	Block, colourless
*V* = 1374.2 (3) Å^3^	0.23 × 0.22 × 0.21 mm
*Z* = 4	

### Data collection

**Table:**

Bruker X8 Proteum diffractometer	2217 independent reflections
Radiation source: Bruker MicroStar microfocus rotating anode	1833 reflections with *I* > 2σ(*I*)
Helios multilayer optics monochromator	*R*_int_ = 0.057
Detector resolution: 10.7 pixels mm^-1^	θ_max_ = 64.4°, θ_min_ = 4.3°
φ and ω scans	*h* = −13→12
Absorption correction: multi-scan (*SADABS*; Bruker, 2013)	*k* = −10→14
*T*_min_ = 0.558, *T*_max_ = 0.614	*l* = −10→12
7001 measured reflections	

### Refinement

**Table:**

Refinement on *F*^2^	Hydrogen site location: inferred from neighbouring sites
Least-squares matrix: full	H atoms treated by a mixture of independent and constrained refinement
*R*[*F*^2^ > 2σ(*F*^2^)] = 0.052	W = 1/[Σ^2^(*F*O^2^) + (0.0762*P*)^2^ + 0.2853*P*] where *P* = (*F*O^2^ + 2*F*C^2^)/3
*wR*(*F*^2^) = 0.130	(Δ/σ)_max_ < 0.001
*S* = 1.09	Δρ_max_ = 0.31 e Å^−^^3^
2217 reflections	Δρ_min_ = −0.50 e Å^−^^3^
159 parameters	Extinction correction: *SHELXL97* (Sheldrick, 2008), FC^*^=KFC[1+0.001XFC^2^Λ^3^/SIN(2Θ)]^-1/4^
0 restraints	Extinction coefficient: 0.173 (7)

### Special details

**Table:**

Geometry. Bond distances, angles *etc*. have been calculated using the rounded fractional coordinates. All su's are estimated from the variances of the (full) variance-covariance matrix. The cell e.s.d.'s are taken into account in the estimation of distances, angles and torsion angles
Refinement. Refinement on *F*^2^ for ALL reflections except those flagged by the user for potential systematic errors. Weighted *R*-factors *wR* and all goodnesses of fit *S* are based on *F*^2^, conventional *R*-factors *R* are based on *F*, with *F* set to zero for negative *F*^2^. The observed criterion of *F*^2^ > σ(*F*^2^) is used only for calculating -*R*-factor-obs *etc*. and is not relevant to the choice of reflections for refinement. *R*-factors based on *F*^2^ are statistically about twice as large as those based on *F*, and *R*-factors based on ALL data will be even larger.

### Fractional atomic coordinates and isotropic or equivalent isotropic displacement parameters (Å^2^)

**Table:**

	*x*	*y*	*z*	*U*_iso_*/*U*_eq_	
O1	−0.28618 (16)	0.3894 (2)	−0.17351 (15)	0.0736 (8)	
N1	0.05732 (13)	0.45578 (15)	−0.22047 (13)	0.0286 (5)	
C1	−0.43665 (19)	0.3624 (2)	−0.5286 (2)	0.0438 (7)	
C2	−0.5574 (2)	0.3438 (2)	−0.6274 (2)	0.0533 (8)	
C3	−0.6620 (2)	0.3275 (2)	−0.5943 (3)	0.0592 (9)	
C4	−0.6478 (2)	0.3307 (2)	−0.4642 (3)	0.0586 (9)	
C5	−0.5288 (2)	0.3513 (2)	−0.3656 (2)	0.0464 (8)	
C6	−0.42153 (17)	0.36703 (19)	−0.39763 (18)	0.0353 (6)	
C7	−0.29561 (18)	0.3890 (2)	−0.28717 (18)	0.0381 (7)	
C8	−0.17901 (16)	0.41077 (19)	−0.31941 (17)	0.0353 (6)	
C9	−0.06310 (17)	0.44018 (19)	−0.19508 (17)	0.0344 (6)	
C10	0.05276 (19)	0.55864 (19)	−0.29863 (19)	0.0378 (6)	
C11	0.1784 (2)	0.5746 (2)	−0.3175 (2)	0.0457 (8)	
C12	0.2928 (2)	0.5773 (2)	−0.1862 (2)	0.0521 (8)	
C13	0.29567 (18)	0.4721 (2)	−0.1097 (2)	0.0500 (8)	
C14	0.17059 (17)	0.4572 (2)	−0.09057 (18)	0.0404 (7)	
Cl1	0.05845 (4)	0.24029 (5)	0.09932 (4)	0.0406 (2)	
H1	−0.36580	0.37180	−0.55080	0.0530*	
H2	−0.56760	0.34230	−0.71580	0.0640*	
H3	−0.74260	0.31430	−0.66040	0.0710*	
H4	−0.71860	0.31900	−0.44240	0.0700*	
H5	−0.51980	0.35480	−0.27770	0.0560*	
H8A	−0.19730	0.47130	−0.38240	0.0420*	
H8B	−0.16000	0.34530	−0.35990	0.0420*	
H9A	−0.08110	0.50800	−0.15790	0.0410*	
H9B	−0.04900	0.38170	−0.13020	0.0410*	
H10A	0.03750	0.62240	−0.25280	0.0450*	
H10B	−0.01790	0.55340	−0.38450	0.0450*	
H11	0.066 (2)	0.393 (2)	−0.268 (2)	0.041 (6)*	
H11A	0.18900	0.51460	−0.37120	0.0550*	
H11B	0.17470	0.64360	−0.36440	0.0550*	
H12A	0.37160	0.58350	−0.20140	0.0620*	
H12B	0.28670	0.64120	−0.13530	0.0620*	
H13A	0.36670	0.47550	−0.02390	0.0600*	
H13B	0.30930	0.40890	−0.15740	0.0600*	
H14A	0.17360	0.38800	−0.04420	0.0480*	
H14B	0.16080	0.51720	−0.03640	0.0480*	

### Atomic displacement parameters (Å^2^)

**Table:**

	*U*^11^	*U*^22^	*U*^33^	*U*^12^	*U*^13^	*U*^23^
O1	0.0501 (9)	0.138 (2)	0.0387 (8)	−0.0265 (11)	0.0239 (7)	−0.0115 (10)
N1	0.0294 (8)	0.0315 (10)	0.0248 (7)	−0.0022 (6)	0.0104 (6)	−0.0011 (7)
C1	0.0371 (10)	0.0478 (15)	0.0450 (11)	−0.0014 (9)	0.0144 (8)	−0.0064 (10)
C2	0.0506 (13)	0.0500 (17)	0.0470 (11)	−0.0009 (11)	0.0057 (9)	−0.0092 (12)
C3	0.0373 (11)	0.0412 (16)	0.0810 (17)	−0.0043 (10)	0.0034 (11)	−0.0122 (13)
C4	0.0343 (11)	0.0499 (17)	0.0903 (18)	−0.0076 (11)	0.0230 (11)	−0.0031 (15)
C5	0.0403 (11)	0.0440 (15)	0.0596 (13)	−0.0058 (10)	0.0245 (9)	−0.0010 (11)
C6	0.0337 (10)	0.0307 (12)	0.0427 (11)	0.0001 (8)	0.0163 (8)	−0.0014 (9)
C7	0.0355 (10)	0.0450 (14)	0.0365 (10)	−0.0033 (9)	0.0171 (8)	−0.0022 (10)
C8	0.0311 (9)	0.0435 (14)	0.0329 (9)	0.0010 (8)	0.0144 (7)	0.0013 (9)
C9	0.0324 (9)	0.0444 (13)	0.0293 (9)	−0.0020 (8)	0.0153 (7)	0.0007 (9)
C10	0.0408 (10)	0.0381 (13)	0.0369 (10)	0.0035 (9)	0.0177 (8)	0.0087 (9)
C11	0.0490 (12)	0.0481 (16)	0.0450 (11)	−0.0047 (10)	0.0239 (9)	0.0090 (11)
C12	0.0430 (12)	0.0569 (18)	0.0565 (13)	−0.0150 (11)	0.0197 (10)	0.0001 (12)
C13	0.0321 (10)	0.0650 (18)	0.0467 (11)	−0.0061 (10)	0.0086 (8)	0.0066 (11)
C14	0.0349 (10)	0.0547 (15)	0.0263 (9)	−0.0078 (9)	0.0063 (7)	0.0054 (9)
Cl1	0.0444 (4)	0.0417 (4)	0.0369 (4)	−0.0004 (2)	0.0171 (2)	0.0073 (2)

### Geometric parameters (Å, º)

**Table:**

O1—C7	1.209 (2)	C2—H2	0.9300
N1—C9	1.503 (3)	C3—H3	0.9300
N1—C10	1.497 (3)	C4—H4	0.9300
N1—C14	1.498 (2)	C5—H5	0.9300
N1—H11	0.95 (2)	C8—H8A	0.9700
C1—C6	1.380 (3)	C8—H8B	0.9700
C1—C2	1.389 (3)	C9—H9A	0.9700
C2—C3	1.379 (4)	C9—H9B	0.9700
C3—C4	1.373 (4)	C10—H10A	0.9700
C4—C5	1.379 (4)	C10—H10B	0.9700
C5—C6	1.400 (3)	C11—H11A	0.9700
C6—C7	1.488 (3)	C11—H11B	0.9700
C7—C8	1.514 (3)	C12—H12A	0.9700
C8—C9	1.518 (3)	C12—H12B	0.9700
C10—C11	1.524 (3)	C13—H13A	0.9700
C11—C12	1.514 (3)	C13—H13B	0.9700
C12—C13	1.514 (3)	C14—H14A	0.9700
C13—C14	1.518 (3)	C14—H14B	0.9700
C1—H1	0.9300		
			
C9—N1—C10	112.23 (16)	C7—C8—H8B	110.00
C9—N1—C14	108.91 (13)	C9—C8—H8A	109.00
C10—N1—C14	111.15 (16)	C9—C8—H8B	109.00
C9—N1—H11	107.3 (15)	H8A—C8—H8B	108.00
C10—N1—H11	109.6 (14)	N1—C9—H9A	109.00
C14—N1—H11	107.4 (13)	N1—C9—H9B	109.00
C2—C1—C6	120.2 (2)	C8—C9—H9A	109.00
C1—C2—C3	119.9 (2)	C8—C9—H9B	109.00
C2—C3—C4	120.3 (2)	H9A—C9—H9B	108.00
C3—C4—C5	120.2 (2)	N1—C10—H10A	109.00
C4—C5—C6	120.1 (2)	N1—C10—H10B	109.00
C5—C6—C7	117.75 (17)	C11—C10—H10A	109.00
C1—C6—C7	123.03 (19)	C11—C10—H10B	109.00
C1—C6—C5	119.22 (19)	H10A—C10—H10B	108.00
O1—C7—C6	120.8 (2)	C10—C11—H11A	109.00
O1—C7—C8	120.36 (19)	C10—C11—H11B	109.00
C6—C7—C8	118.87 (16)	C12—C11—H11A	109.00
C7—C8—C9	110.77 (15)	C12—C11—H11B	109.00
N1—C9—C8	112.86 (14)	H11A—C11—H11B	108.00
N1—C10—C11	110.89 (18)	C11—C12—H12A	110.00
C10—C11—C12	111.59 (17)	C11—C12—H12B	110.00
C11—C12—C13	109.56 (19)	C13—C12—H12A	110.00
C12—C13—C14	110.85 (19)	C13—C12—H12B	110.00
N1—C14—C13	111.44 (15)	H12A—C12—H12B	108.00
C2—C1—H1	120.00	C12—C13—H13A	109.00
C6—C1—H1	120.00	C12—C13—H13B	109.00
C1—C2—H2	120.00	C14—C13—H13A	109.00
C3—C2—H2	120.00	C14—C13—H13B	110.00
C2—C3—H3	120.00	H13A—C13—H13B	108.00
C4—C3—H3	120.00	N1—C14—H14A	109.00
C3—C4—H4	120.00	N1—C14—H14B	109.00
C5—C4—H4	120.00	C13—C14—H14A	109.00
C4—C5—H5	120.00	C13—C14—H14B	109.00
C6—C5—H5	120.00	H14A—C14—H14B	108.00
C7—C8—H8A	110.00		
			
C10—N1—C9—C8	69.9 (2)	C4—C5—C6—C7	180.0 (2)
C14—N1—C9—C8	−166.55 (18)	C1—C6—C7—O1	−178.0 (2)
C9—N1—C10—C11	177.56 (15)	C1—C6—C7—C8	1.9 (3)
C14—N1—C10—C11	55.3 (2)	C5—C6—C7—O1	2.4 (4)
C9—N1—C14—C13	179.67 (18)	C5—C6—C7—C8	−177.6 (2)
C10—N1—C14—C13	−56.2 (2)	O1—C7—C8—C9	−4.6 (3)
C6—C1—C2—C3	−1.4 (4)	C6—C7—C8—C9	175.5 (2)
C2—C1—C6—C5	0.9 (4)	C7—C8—C9—N1	176.49 (18)
C2—C1—C6—C7	−178.7 (2)	N1—C10—C11—C12	−56.2 (2)
C1—C2—C3—C4	0.7 (4)	C10—C11—C12—C13	56.4 (2)
C2—C3—C4—C5	0.6 (4)	C11—C12—C13—C14	−56.6 (2)
C3—C4—C5—C6	−1.2 (4)	C12—C13—C14—N1	57.1 (2)
C4—C5—C6—C1	0.4 (4)		

### Hydrogen-bond geometry (Å, º)

**Table:**

*D*—H···*A*	*D*—H	H···*A*	*D*···*A*	*D*—H···*A*
N1—H11···Cl1^i^	0.95 (2)	2.15 (2)	3.0837 (18)	171 (2)
C4—H4···Cl1^ii^	0.93	2.82	3.745 (3)	172
C14—H14*B*···O1^iii^	0.97	2.45	3.249 (3)	139

Symmetry codes: (i) *x*, −*y*+1/2, *z*−1/2; (ii) *x*−1, −*y*+1/2, *z*−1/2; (iii) −*x*, −*y*+1, −*z*.
